# Review of Recent Advances in Lipid Analysis of Biological Samples via Ambient Ionization Mass Spectrometry

**DOI:** 10.3390/metabo11110781

**Published:** 2021-11-15

**Authors:** Haiyan Lu, Hua Zhang, Shuling Xu, Lingjun Li

**Affiliations:** 1 School of Pharmacy, University of Wisconsin-Madison, Madison, WI 53705, USA; hlu244@wisc.edu (H.L.); hzhang775@wisc.edu (H.Z.); sxu374@wisc.edu (S.X.); 2 Department of Chemistry, University of Wisconsin-Madison, Madison, WI 53706, USA

**Keywords:** lipidomics, structural elucidation, ambient ionization mass spectrometry, biological samples

## Abstract

The rapid and direct structural characterization of lipids proves to be critical for studying the functional roles of lipids in many biological processes. Among numerous analytical techniques, ambient ionization mass spectrometry (AIMS) allows for a direct molecular characterization of lipids from various complex biological samples with no/minimal sample pretreatment. Over the recent years, researchers have expanded the applications of the AIMS techniques to lipid structural elucidation via a combination with a series of derivatization strategies (e.g., the Paternò–Büchi (PB) reaction, ozone-induced dissociation (OzID), and epoxidation reaction), including carbon–carbon double bond (C=C) locations and sn-positions isomers. Herein, this review summarizes the reaction mechanisms of various derivatization strategies for C=C bond analysis, typical instrumental setup, and applications of AIMS in the structural elucidation of lipids from various biological samples (e.g., tissues, cells, and biofluids). In addition, future directions of AIMS for lipid structural elucidation are discussed.

## 1. Introduction

**Figure 1:**
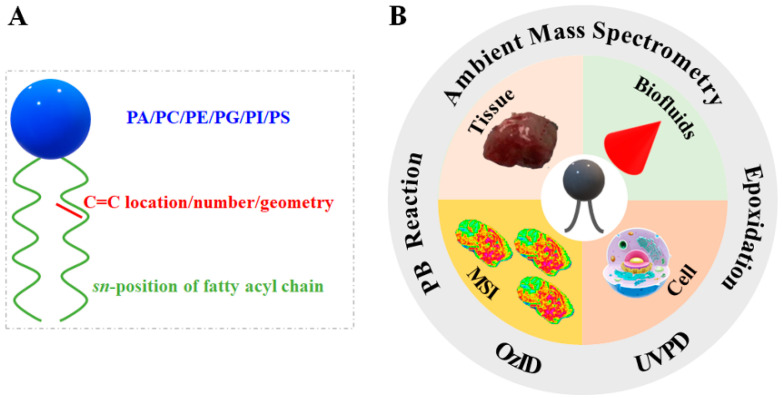
(**A**) The schematic figure indicates the chemical structure diversity and complexity of unsaturated lipids; (**B**) representation of different derivatization strategies coupling of AIMS techniques (outer ring) and examples of their applications (central pie chart).

Lipids are essential biomolecules involved in various cellular functions, including structural components, energy reservoirs, and signal molecules in biological systems [1,2]. Unsaturated lipids, as a subclass of lipids, structurally differ in the types of headgroups (such as phosphatidic acids (PA), phosphatidylcholine (PC), phosphatidylethanolamine (PE), phosphatidylglycerol (PG), phosphatidylinositol (PI), and phosphatidylserine (PS)), carbon–carbon double bond (C=C) locations, numbers, as well as cis/trans geometric isomerism, and the sn-position of the fatty acyl chain (Figure 1A), which complicate structural characterization [3,4]. In particular, the biological functions of lipids highly depend on their structural diversity and varying expression levels [5]. For example, the unsaturation level of lipids affects cell physiological properties (e.g., the permeability and fluidity of the cell membrane, cardiolipin remodeling, and neurotransmitter release) [6,7]. Metabolic dysregulations of lipids C=C bond positional isomers, as well as sn-positions, may be related to many diseases, such as cancers, type II diabetes (T2D), Alzheimer’s, and other neurodegenerative diseases [8,9,10,11]. Therefore, precise lipid structural elucidation in complex biological samples is of great significance for studying the fundamental mechanism of lipid metabolism and the pathogenesis of various diseases [7].

Mass spectrometry (MS) is widely used for the identification and quantitation of lipids from biological samples with a high sensitivity and specificity [12,13,14]. Generally, tandem mass spectrometry (MS/MS) via the collision-induced desorption (CID) method can provide a lipid head group and fatty acyl chain composition, but has limited application in directly locating the C=C positions, because low energy CID cannot obtain informative fragment ions to assign the C=C location [15,16,17]. Hence, emerging MS-based dissociation methods, including ozone-induced dissociation (OzID) [18,19], electron-induced dissociation (EID) [20], ultraviolet photodissociation (UVPD) [1,21], and selective C=C bond derivatization methods (e.g., the Paternò–Büchi (PB) reaction [22,23], meta-chloroperoxybenzoic acid (*m*-CPBA) epoxidation [24,25], and peracetic acid (PAA) epoxidation [5]), have been developed to elucidate the lipid structure. Traditionally, MS coupled with chromatographic approaches (such as liquid chromatography (LC)) are effective tools for the characterization of lipid isomers [26,27,28], but these methods usually require tedious sample preparations prior to MS analysis. Compared with traditional MS approaches, ambient ionization mass spectrometry (AIMS) techniques allow for the rapid and direct analysis of lipids from raw complex biological samples with minimal or no sample treatments [29].

For example, lipids from various human cancerous tissue samples have been directly characterized by AIMS techniques, including desorption electrospray ionization mass spectrometry (DESI-MS) [30], rapid evaporative ionization mass spectrometry (REIMS) [31], MasSpec Pen [32], and internal extractive electrospray ionization mass spectrometry (iEESI-MS) [33,34]. Atmospheric pressure matrix-assisted laser/desorption ionization mass spectrometry (AP-MALDI-MS) is an alternative AIMS technique that enables the obtaining of high spatial resolution images of lipids in the brain at an atmospheric pressure condition [35]. Compared with conventional vacuum matrix-assisted laser desorption/ionization mass spectrometry (MALDI-MS) [36,37], AP-MALDI-MS significantly increases the ease of sample preparation [38,39]. The significant difference between AIMS techniques and the conventional high vacuum MALDI-MS is that AIMS techniques offer minimal sample preparation and effectively eliminate the matrix-associated limitations with no need of using the exogenous matrix compound [40]. Taken together, these studies laid a solid foundation for the applications of AIMS techniques in advanced lipidomic research. Therefore, a series of AIMS techniques have been developed with expanded applications in lipid structural elucidation (including C=C locations and sn-positions) over the past few years. Herein, this review summarizes the mechanisms of various derivatization strategies for C=C location analysis, typical instrumental setup, and applications of AIMS in the structural elucidation of lipids from various biological samples within the past five years. The applications of AIMS techniques coupling with different derivatization strategies in the lipid structural elucidation in this review are illustrated in Figure 1B.

## 2. Mechanism and Instrumental Setup of Various Strategies for Lipid Structural Elucidation

Numerous AIMS techniques have been applied for the characterization of C=C locations, as well as sn-positions, in unsaturated lipids via combining with different types of derivatization strategies, including (1) PB reaction, (2) OzID and UVPD, (3) epoxidation reaction and (4) other strategies. In this section, both the mechanism between various derivatization strategies and C=C bond analysis and the typical instrumental setup of AIMS techniques coupled with different derivatization strategies for lipid structural elucidation will be introduced and discussed.

### 2.1. PB Reaction

**Figure 2:**
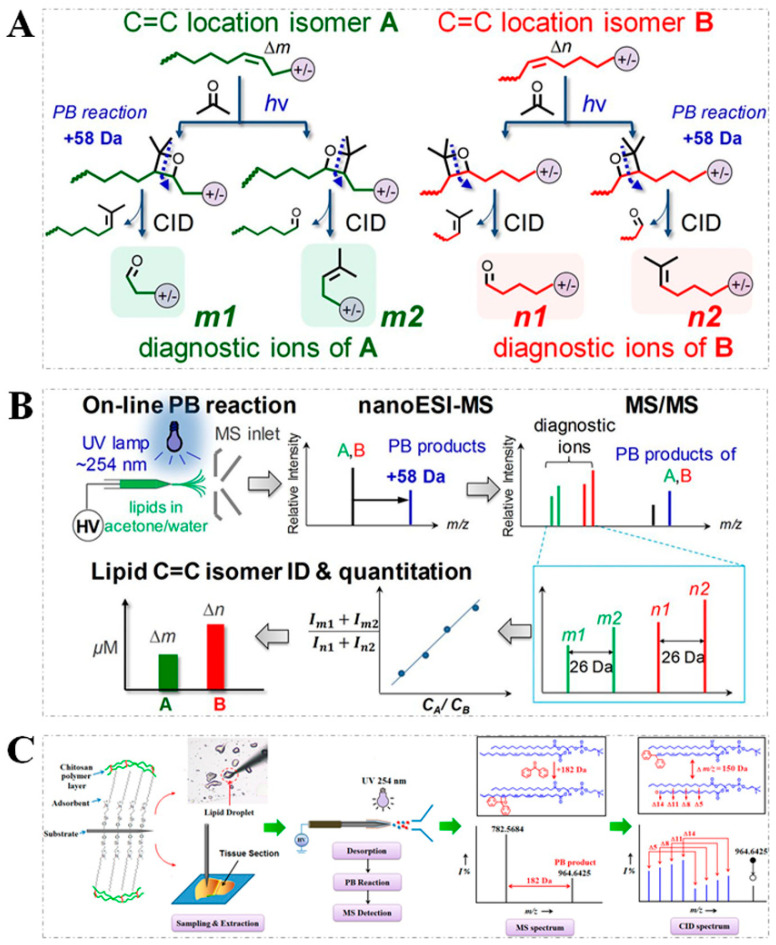
(**A**) The formation mechanism of C=C diagnostic ions from lipid C=C location isomers via PB reaction; (**B**) the workflow of direct characterization and quantitation of lipid C=C location isomers by PB−-nanoESI−MS/MS (note: Figure 2A,B reproduced from Ref. [16] with permission from National Academy of Sciences); (**C**) the workflow of SCP−PB−nanoESI−MS method for in vivo and microscale profiling of lipid characterization in tissue sections (reproduced permission from Ref. [47]. Copyright (2019) American Chemical Society).

The PB reaction is a classic [2 + 2] photochemical reaction and is widely used for the formation of oxetane ring compounds in organic synthesis [41]. The PB reaction has gained attention for lipid structural elucidation, since its coupling to tandem MS (PB-MS/MS) analysis can result in C=C positional information in unsaturated lipids [42,43]. To clearly describe the reaction mechanism of the PB reaction with the C=C bond, acetone as a PB reagent is taken as an example. As shown in Figure 2, for each lipid C=C location isomer, two regioisomers of the PB products (with 58 Da mass shift from the original unsaturated lipids) could form, owing to two possible orientations for the addition of acetone to a C=C bond, thereby generating a pair of diagnostic ions that can be used for assigning (Figure 2A) and quantifying (Figure 2B) the C=C location isomers of unsaturated lipids [16]. Despite great progress being reported in using acetone as a derivatization reagent in the PB reaction, other PB reagents (e.g., benzophenone [44], 2-acetylpyridine (2-AP) [26], and 2′,4′,6′-trifluoroacetophenone (triFAP) [45]) have been developed for the study of unsaturated lipids. It has been noted that the specific mass shift of PB products is related with the PB reagents mass. PB-MS/MS have several notable features, including the fact that (1) the specific mass increase from the original unsaturated lipids can be readily used for the identification of PB products, (2) the diagnostic ions derived from the PB products enable confident identifications of the C=C location isomers, (3) the ion intensities of diagnostic ions could be used for the quantitative analysis of lipid C=C location isomers, and (4) there is a simple experimental setup for the reaction, and no need for MS instrument modification. With these advantages, there have been some derivatization methods based on the PB reaction. For example, a double derivatization strategy, which utilized both an acetone labeling of the C=C bond via the PB reaction in order to provide diagnostic ions for pinpointing the C=C locations and a *N*,*N*-diethyl-1,2-ethanediamine (DEEA) labeling of the carboxyl group in order to improve the MS response of diagnostic ions, was developed for the sensitivity localization of the C=C bond in free fatty acids (FFAs) [46].

Additionally, to allow the PB reaction to be available for the characterization of C=C locations in unsaturated lipids from broad types of samples, different sampling methods have been proposed. For instance, a polymer coating transfer enrichment (PCTE) method coupled with the PB reaction was developed for a nanoelectrospray ionization mass spectrometry (PCTE-PB-nanoESI-MS) analysis of unsaturated lipids in human biofluid samples [48]. Similarly, the in vivo and microscale profiling of lipid C=C location isomers in biological tissues has been realized by combining the PB reaction with surface-coated probe nanoESI-MS (SCP-PB-nanoESI-MS) [47]. In SCP-PB-nanoESI-MS analysis (Figure 2C), a biocompatible SPEM probe with a nanospray tip was used for the extraction and ionization of lipids from a biological tissue sample, and then the solvent containing the PB regents was used to desorb lipids enriched on the SPEM probe. Subsequently, the PB reaction was triggered by irradiation with an UV light at 254 nm. After that, the high voltage was applied on the SPEM probe for nanoESI-MS/MS and generated diagnostic ions corresponding to the location of the C=C bond.

### 2.2. OzID and UVPD

**Figure 3:**
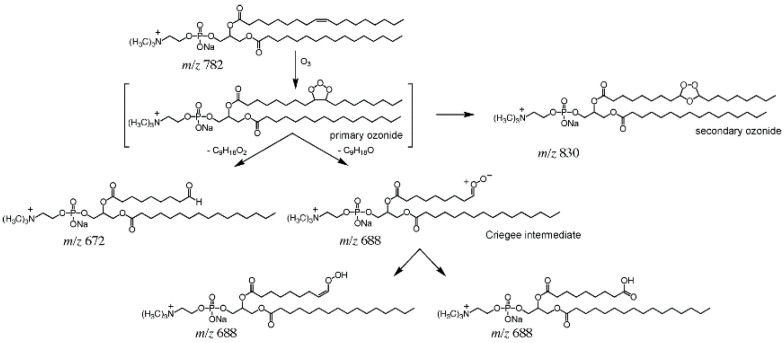
The reaction mechanism of OzID with mass−selected [GPCho (16:0/9Z−18:1) + Na]^+^ ions (*m*/*z* 782) in ion trap mass spectrometer (Reproduced permission from Ref. [49]. Copyright (2008) American Chemical Society).

OzID is an ion activation method that exploits the gas-phase reaction between mass-selected unsaturated lipid ions and ozone, which could selectively cleave the C=C bond located on the acyl chains, thereby generating diagnostic fragment ions (such as aldehyde and Criegee ions) to assign sites of the C=C bond in unsaturated lipids. Taking GPCho (16:0/9Z-18:1) as an example, the mechanism of OzID with unsaturated lipid ions in an ion trap mass spectrometer was shown in Figure 3. The mass-selected sodium adduct ion of GPCho (16:0/9Z-18:1) at *m*/*z* 782 could produce primary and secondary ozonide under the presence of ozone, and could then generate two product ions at *m*/*z* 672 and *m*/*z* 688 corresponding to aldehyde and Criegee ions, which were used for the identification of C=C positions, caused by a neutral loss of 110 Da and 94 Da from primary ozonide under CID analysis, respectively [49]. Additionally, UVPD, as a high-energy photoactivation method, could provide informative lipid structural information, which has been used for the analysis of different classes of lipids, either as a standalone MS/MS strategy or in conjunction with a collisional activation approach to develop hybrid MS^n^ strategies. In essence, UVPD allows for the identification of C=C locations based on characteristic diagnostic ions with 24 Da mass differences resulting from the cleavage of carbon–carbon bonds adjacent to the C=C bond [21,50,51,52]. Both OzID and UVPD are of great potential for lipid structural analysis, but they often require special instrument modification.

### 2.3. Epoxidation Reaction

**Figure 4:**
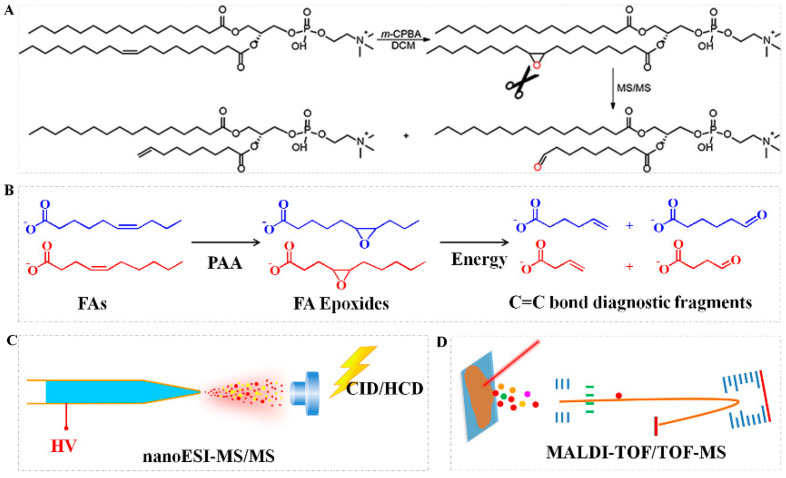
The mechanism of epoxidation reaction induced by *m*−CPBA (**A**) (reproduced permission from Ref. [24]. Copyright (2019) American Chemical Society.) and PAA (**B**) to produce diagnostic fragment ion pair. The workflow of PAA epoxidation in combination with nanoESI−MS/MS (**C**) for assigning C=C bond in FAs from solution and MALDI−TOF/TOF−MS (**D**) for spatial characterization of C=C bond in FAs from tissue sections (note: Figure 4B–D, reproduced from Ref. [5] with permission from the Royal Society of Chemistry).

The epoxidation reaction is another chemical derivatization strategy for the structural determination of lipids. Epoxidation using either meta-chloroperoxybenzoic acid (*m*-CPBA) (Figure 4A) [24] or peracetic acid (PAA) (Figure 4B) [5] coupled with CID-MS/MS to produce a diagnostic ion pair, as shown in Figure 4. Briefly, unsaturated lipids are firstly oxidized via *m*-CPBA or PAA to generate an epoxide; then, the epoxide product is isolated for CID-MS/MS analysis, which generates a pair of diagnostic ions for pinpointing the C=C bond location [24,25]. PAA epoxidation can be combined with nanoESI-MS/MS for assigning the C=C bond in fatty acids (FAs) from the solution (Figure 4C). The method attained satisfactory linearity in a quantitative analysis of C=C bond positional FA isomers (including FA 16:1 (9Z), FA 18:1 (9Z), and FA 18:1 (11Z)) (R^2^ > 0.9989), the limit of detections (LODs) and relative standard deviations (RSDs) for three tested FAs were 4.2 nM–5.6 nM and 3.4–8.9%, respectively. Furthermore, PAA epoxidation can be combined with matrix-assisted laser desorption/ionization time-of-fight/time-of-fight MS (MALDI-TOF/TOF-MS) for the spatial mapping and characterization of the C=C bond in FAs from tissue sections (Figure 4D) [5]. Although epoxidation reactions by both *m*-CPBA and PAA have been developed for the analysis of unsaturated lipids, PAA offers the advantage of an easy sample clean-up, owing to the high volatility of PAA, and PAA presents minimal potential side effects, such as ion suppression and MALDI matrix oxidization, during on-tissue epoxidation [5]. Recently, chloroauric acid (HAuCl_4_) was added into an electrospray solvent during microdroplet mass spectrometry analysis; the resulting oxidation products could be used to identify the location of one or more C=C bonds in unsaturated lipids. The mechanism of this method involved C=C bond epoxidation, followed by the formation of the final products [53].

### 2.4. Other Strategies

**Figure 5:**
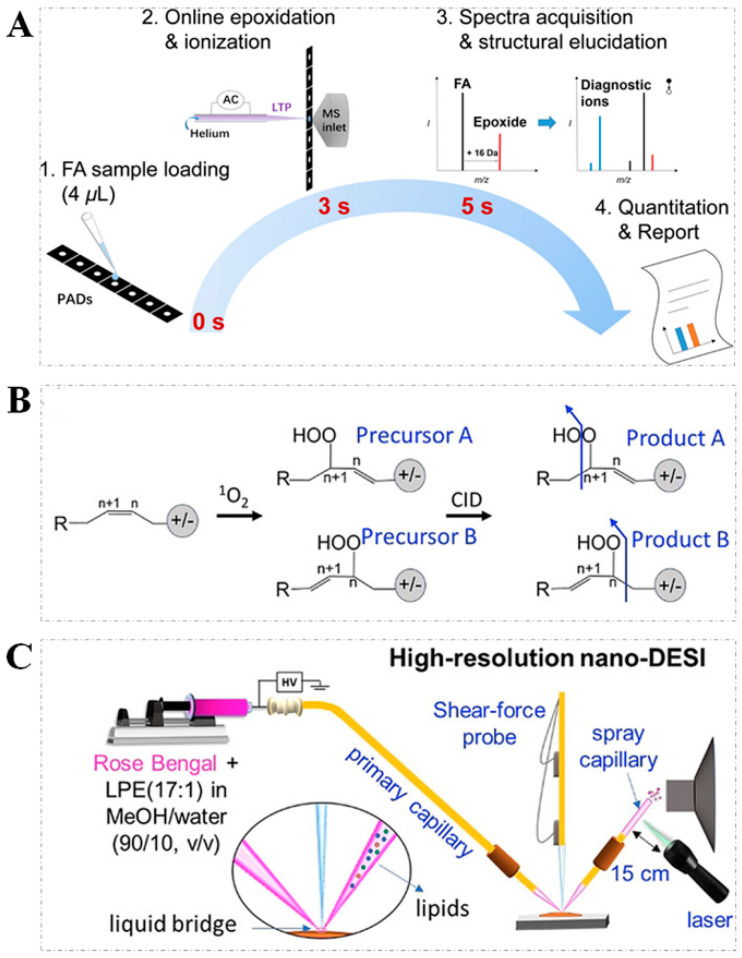
(**A**) The workflow of online epoxidation/ionization of unsaturated FAs on paper strips by LTP−MS (reproduced permission from Ref. [55]. Copyright (2018) American Chemical Society); (**B**) the mechanism of C=C bond oxidation by ^1^O_2_ and fragmentation via CID to yield products; (**C**) the experimental setup of online ^1^O_2_ derivatization of C=C bond combined with nano−DESI−MS (note: Figure 5B,C, reproduced from Ref. with permission from permission of Wiley-VCH GmbH).

In addition to the PB reaction, OzID, UVPD, and epoxidation derivatization using *m*-CPBA, PAA, as well as HAuCl_4_, plasma, and electrochemical-induced epoxidations were also developed in the structural lipidomic study. For instance, the C=C bond can be oxidized and cleaved under the low-temperature plasma (LTP) condition, and the generated fragment ions can be used to assign C=C positions. In LTP-induced epoxidation, air was used as the oxidizing agent, with no need for other special solvents [54]. Moreover, the LTP probe enabled the online epoxidation and rapid analysis of the monounsaturated, and polyunsaturated FAs on paper-based analytical devices (PADs) within 2 s (Figure 5A) [55]. In this workflow, when the paper strip containing the unsaturated FAs solution was placed between a LTP probe and the MS ion source inlet, the epoxidation reaction was promptly initiated, and then the epoxidation product was immediately ionized and detected by MS when the paper strip was in touch with the MS ion source inlet. NanoESI-MS, as the variant of traditional ESI-MS, offers a special advantage of ESI-MS in the analysis of a broad range of biomolecules with low sample consumption [56,57]. An on-demand electrochemical epoxidation coupled with a nanoESI-MS strategy was developed to locate the C=C bond position in lipids [58]. In this workflow, the onset of the electro-epoxidation of the C=C bond could be controlled by tuning the voltage, and the diagnostic ions derived from the fragmentation of electro-epoxidized products by MS/MS were used to indicate the C=C positions. To circumvent the challenge in spatially resolving positional isomers in lipidomic analyses, the method for the online selective photochemical derivatization of the C=C bond by singlet oxygen (^1^O_2_) was proposed [2]. The mechanism of C=C bond oxidation by ^1^O_2_ (Figure 5B) can be briefly descripted as follows. ^1^O_2_ generated under a photosensitizer interacts with unsaturated lipids to form lipid hydroperoxides (LOOHs), where the cleavage of LOOHs at the location of the hydroperoxide group in CID analysis indicates the C=C locations. Nanospray desorption electrospray ionization mass spectrometry (nano-DESI-MS), as a variant of DESI-MS, showed its advantages in the high-efficiency extraction and quantitative determination of phospholipids [59,60]. Figure 5C shows the experimental setup of the online ^1^O_2_ derivatization of the C=C bond combined with nano-DESI-MS. Moreover, a combined approach of cryogenic gas-phase infrared spectroscopy, ion mobility-mass spectrometry (IM-MS), and quantum chemical calculations for the investigation of the C=C positional in FAs isomers was implemented by a non-covalent formation without chemical treatments or instruments modifications [61].

In summary, among the above-mentioned derivatization methods (such as the PB reaction, OzID, UVPD, epoxidation by *m*-CPBA, PAA and HAuCl_4_, plasma oxidation, electrochemical epoxidation, and ^1^O_2_), the PB reaction is the most classic photochemical strategy used for pinpointing C=C location isomers of unsaturated lipids in both shotgun analysis [16,23] and high-performance liquid chromatography-tandem mass spectrometry (HPLC-MS/MS) [7,8,45] workflow. UVPD can locate C=C positions and sn-specific fatty acyls, whereas OzID has been reported for locating C=C positions and is used primarily for the sodium-adducted lipids in shotgun-based lipidomics [62,63]. The common concern for the PB reaction, OzID, and UVPD is that the use of UV light/ozone in these reactions might pose unexpected potential health risks [3,64]. Unlike the PB reaction, UVPD, and OzID, the epoxidation reaction induced by *m*-CPBA, PAA, and HAuCl_4_ offers a much higher specificity and occurs under ambient conditions without UV irradiation or hardware modification [65]. For LTP oxidation and on-demand electrochemical epoxidation, they provide alternative approaches to determine C=C locations without explicit chemical derivatization or sample treatment prior to MS detection, which might hold potential for high-throughput lipid structural elucidation.

## 3. The Applications of AIMS in Lipid Structural Elucidation

The C=C location, sn-position, and branched chain isomers in unsaturated lipids have an important influence on their biological functions. Therefore, precise lipid structural characterization is critical for in-depth lipidomic investigation. In this section, we introduce the applications of AIMS techniques in the structural elucidation of lipids from different types of biological samples (e.g., tissues, biofluids, and cells) within the past five years.

### 3.1. Direct Characterization of Unsaturated Lipid Isomers in Tissue Samples

A tissue specimen is one of the most common biological samples for both clinical and laboratory study; in particular, the fine structure characterization of unsaturated lipids in tissue samples plays a critical role in disease molecular diagnosis [66]. The PB reaction, as a typical photochemical derivatization method, has been coupled with AIMS techniques for the direct characterization of the C=C location in tissue samples. For example, online PB reaction coupling with nanoESI-MS/MS enabled the identification of 96 unsaturated FAs and glycerophospholipids (GPs) from rat brain tissue, and the relative quantitation of C=C positional isomers in FAs with chain lengths ranging from 16 to 22 carbons. Based on the intensities of the diagnostic ion, the relative abundances of Δ11 C=C location isomers from FA (18:1), PC (18:0–18:1), and PC (18:1–18:1) between normal and cancerous mouse breast tissues were compared, offering a new perspective for the precise molecular diagnosis of malignancies [16].

**Figure 6:**
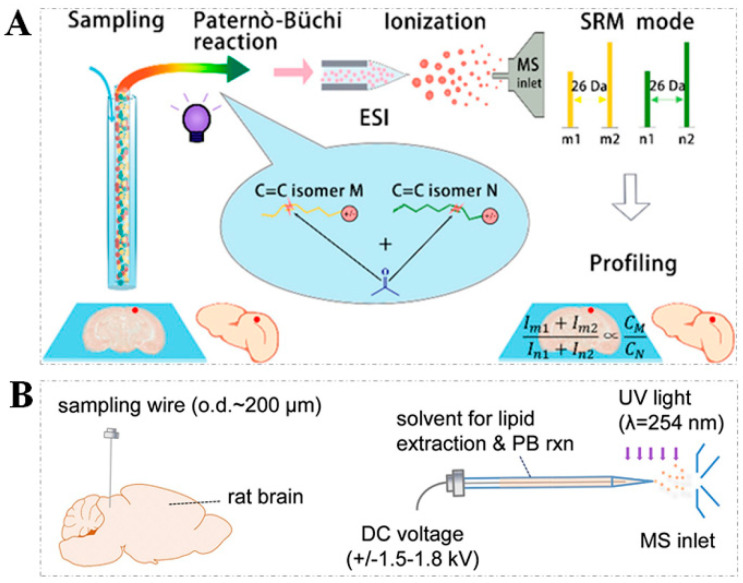
The application examples of typical AIMS techniques in pinpointing C=C locations via PB reaction. (**A**) Direct sampling of tissue section by LMJ-SSP (reproduced permission from Ref. [67]. Copyright (2018) American Chemical Society); (**B**) tissue sampling by a stainless-steel wire (reproduced from Ref. [68] with permission from Elsevier B.V.).

In addition, an in situ lipid extraction and ionization AIMS technique by combining the PB reaction with a liquid microjunction surface sampling probe (LMJ-SSP) (Figure 6A) was developed for the identification and quantitation of lipid C=C location isomers [67], with fast analysis (<2 min), good reproducibility (RSD < 10%), and a satisfactory linear relationship (R^2^ = 0.999). By exploiting this method, unsaturated lipid C=C bond isomers in the tissue sections of the rat brain, lung, liver, spleen, and kidney were rapidly profiled, showing great potential in rapid tissue analysis and clinical molecular diagnosis. As an example, a biopsy-type sampling method combined with the PB reaction was used for the direct sampling and analysis of lipids from rat organs (including the brain, kidney, and liver) (with an area of approximately 0.008 cm^2^ for each analysis) (Figure 6B) [68]. A total of 15 unsaturated FAs and 31 unsaturated phospholipids, including phosphatidylserines (PSs), phosphatidylglycerols (PGs), phosphatidylinositols (PIs), phosphatidylethanolamines (PEs), phosphatidic acids (PAs), lyso-PAs (LPAs), and lyso-PGs (LPGs), were successfully identified. Furthermore, investigations of lipid compositions and C=C location isomers from human intestinal tissue and zebrafish were successfully accomplished through SCP-PB-nanoESI-MS [47].

Lipids have a substantial influence on vertebrate embryogenesis. The large-scale and spatiotemporal monitoring of unsaturated lipids with C=C bond specificity in zebrafish embryos has been achieved by PB-nanoESI-MS/MS [69]. The findings indicated that the lipid isomer composition remained to be stable in yolk throughout embryogenesis, while obvious alterations in the ratios of C=C location and fatty acyl composition isomers for some lipids were revealed between blastomeres and yolk from the zygote to 4 h post-fertilization. Moreover, the double derivatization strategy based on the PB reaction has enabled the assignment of the C=C locations of FFAs from nonalcoholic fatty liver disease (NAFLD) mouse model and control mouse liver samples [46]. Infrared matrix-assisted laser desorption electrospray ionization (IR-MALDESI), as a hybrid soft ionization source based on IR-laser desorption followed by electrospray post-ionization, has been combined with on-tissue *m*-CPBA epoxidation for the in situ detection of FAs C=C positional isomers in rat liver and human bladder cancer tissue [65]. Furthermore, when coupling the ^1^O_2_ reaction with nano-DESI-MS, several isomeric lipids from mouse muscle and uterine tissues were directly detected [2]. Recently, an online coupling of the PB reaction and radical-direct fragmentation with in-capillary extraction nanoelectrospray ionization mass spectrometry (ICE-nanoESI-MS) [70] was developed in order to determine C=C locations and stereospecific numbering positions of potential lipid biomarkers for human colorectal cancer under an ambient condition; the results indicated that lysophospholipids could act as potential biomarkers related to the pathogenesis of human colorectal cancer. In short, these results confirmed that AIMS, combined with derivatization methods, could successfully detect the molecular variations in unsaturated lipid isomers within tissue samples, showing the potential capability in providing deeper insights into the processes resulting from an altered unsaturated lipid metabolism within complex tissues.

### 3.2. Direct Characterization of Unsaturated Lipid Isomers in Biofluid Samples

**Figure 7:**
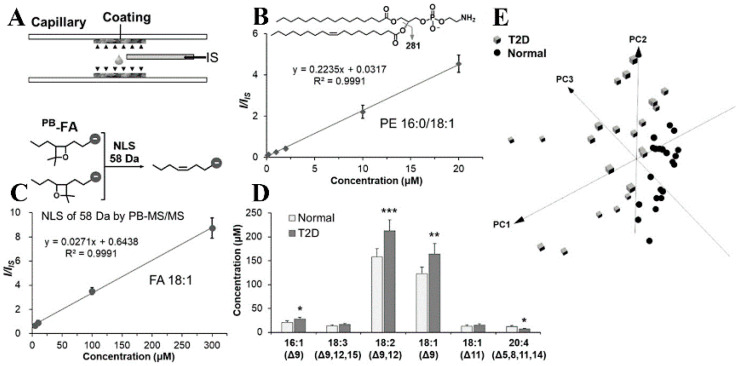
Quantitation of lipids in biofluid samples by PCTE−PB−nanoESI−MS (reproduced from Ref. [48] with permission from Wiley-VCH Verlag GmbH & Co. KGaA, Weinheim). (**A**) Incorporation of internal standards by polymer coating; (**B**) calibration curve of PE 16:0/18:1; (**C**) calibration curve of FA 18:1. Inset shows the fragmentation of derivatized FAs; (**D**) comparison of absolute concentration of some major free FAs in normal and T2D blood samples; (**E**) 3D PCA plot of the data of major free FAs in normal and T2D blood samples. (Note: neutral loss scan (NLS), type 2 diabetes (T2D), * *p* < 0.05, ** *p* < 0.01, *** *p* < 0.001, Student’s *t*-test).

Besides tissue samples, AIMS techniques in combination with derivatization strategies are also widely used for lipidomic studies from biofluid samples, such as plasma, blood, and serum. For example, PB-MS/MS enabled the quantitation of unsaturated FAs from blood, and from plasma in a much more rapid manner [71]. Excitingly, the direct MS quantitative and qualitative determination of the lipid structure at the C=C positions level in small volume biofluid samples (5 μL) has been achieved (Figure 7) by PCTE-PB-nanoESI-MS [48]. Despite the capability of the PCTE-PB-nanoESI-MS method in identifying potential blood biomarkers for T2D being demonstrated, the reproducibility of the quantitation of lipids in the biofluid sample needs to be further improved because the current PCTE procedure still requires a multi-step manual transfer of samples and solvents. The unsaturated lipid oxidation facilitated by LTP has enabled the identification and quantitation of C=C locations in FAs within human plasma samples [72]. In a similar manner, locating the C=C bond in unsaturated phospholipids (e.g., phosphatidylcholines (PCs), PEs, and phosphatidylinositols (PIs)) from bovine liver extract has been performed [73], which further extended the LTP epoxidation for the analyses of a large variety of phospholipids. Moreover, the LTP probe enabled the online epoxidation and rapid identification of unsaturated FA isomers in human, equine, and fetal bovine serum on PADs within 2 s. The LOD was determined to be 0.1 μM for oleic acid. The method shows great potential in point-of-care (POC) clinical diagnosis with fast and high-throughput features [55]. Unlike LTP oxidation, an integrated electrocatalytic nESI-MS platform that utilizes non-inert metal electrodes (e.g., Ir and Ru) has achieved a rapid quantification of FAs isomers from raw serum sample [74].

### 3.3. Direct Characterization of Unsaturated Lipid Isomers in Cell Samples

The cell is the basic unit of living organisms and is responsible for many life processes. Monitoring quantitative changes in metabolites (e.g., FAs and PCs) in cell samples has received increasing attention to facilitate studies in cancer biology [75,76]. The quantitative characterization of FAs isomers from complex biological samples has been achieved by using PAA-induced epoxidation coupled with nanoESI-MS, offering a broad utility for various MS platforms. The method enabled confident identifications of 37 monounsaturated FAs with C=C bond positions from human cell lines (including HPV16-E6E7 immortalized human pancreatic duct epithelial (HPDE/E6E7) cells and pancreatic cancer cells (PANC-1)) [5]. Additionally, the selective detection of unsaturated FAs from normal and cancerous human prostate cells has been realized by PB-MS/MS, enabling a better understanding of the role of the FA isomers in the development of human prostate cancer [71]. In recent years, single cell analysis has attracted increasing attention, with a capability to allow for the study of cell-to-cell heterogeneity and to facilitate novel biomarker discovery [77]. A simple single cell mass spectrometry (SCMS) technique combined with the PB reaction was developed [6], and achieved the determination of C=C locations in unsaturated lipids at the single-cell level, showing the potential utility for other reactive SCMS studies of the molecular characterization of unsaturated lipids in single cells.

### 3.4. Spatial Visualization of Unsaturated Lipid Isomers within Tissue Sections

**Figure 8:**
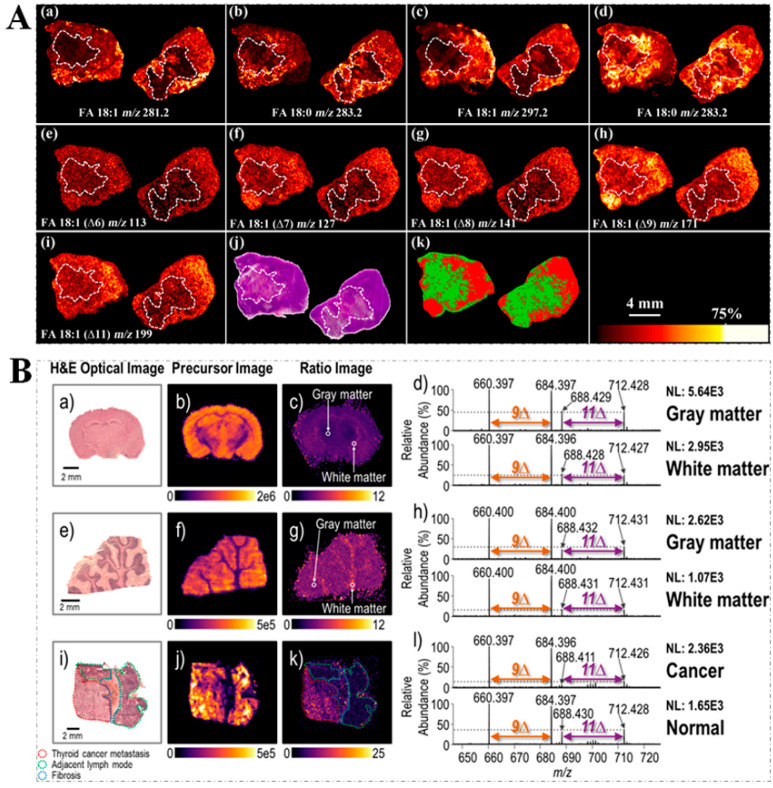
(**A**) The on-tissue PAA epoxidation strategy combined with MALDI-TOF/TOF-MS platform to map the spatial distribution of FA isomers in tissue sections. Full-MS images of (**a**) FA 18:1 and (**b**) FA 18:0 without epoxidation as well as (**c**) epoxy-FA 18:1 and (**d**) FA 18:0 after on-tissue epoxidation. MALDI-TOF/TOF-tandem MS images of (**e**) FA 18:1 (D6), (**f)** FA 18:1 (D7), (**g**) FA 18:1 (D8), (**h**) FA 18:1 (D9), (**i**) FA 18:1 (D11). (**j**) The H&E stained histological image. (**k**) Segmentation pipeline analysis of the MS^2^ data: region rich in cancer cells (red) and region containing few cancer cells with necrosis (green). The left side tissue section was from untreated sample while the right side tissue section was from sample treated with radiation in all the images. (reproduced from Ref. [5] with permission from the Royal Society of Chemistry); (**B**) DESI−MS coupled with the 193 nm UVPD for characterization of phospholipid isomers in tissue sections. H&E optical images of different tissue sections, including (**a**) mouse brain, (**e**) human brain, and (**i**) lymph node with thyroid cancer metastasis. DESI-MS ion image of *m*/*z* 798 in different tissue sections, including (**b**) mouse brain, (**f**) human brain, and (**j**) lymph node with thyroid cancer metastasis. (**c**) DESI-UVPD ratio image of the ratio of the summed intensities of the UVPD double-bond diagnostic ions (I_*m*/*z* 660 + 684_)/(I_*m*/*z* 688 + 712_), (**g**) DESI ratio image of the ratio of the summed intensities of the UVPD double-bond diagnostic ions (I_*m*/*z* 660 + 684_)/(I_*m*/*z* 688 + 712_), and (**k**) DESI intensity ratio image of (I_*m*/*z* 684_/I_*m*/*z* 712_). Expanded region of UVPD mass spectra of (**d**) the white and gray matter, (**h**) the white and gray matter, and (**l**) cancerous and normal parts of tissue. (reproduced permission from Ref. [87]. Copyright (2018) American Chemical Society).

Mass spectrometry imaging (MSI) allows for the molecularly mapping of the spatial distribution of compounds over the sample, and provides multiplexed information without the need for labeling or staining, which has emerged as a promising technique in many areas (e.g., biomedical science, food science, and forensics) [78,79,80,81]. Traditionally, MSI often reflects the summed distribution of multiple isomeric molecules. Nevertheless, characterizing lipids only at the sum of the compositional level easily neglects lipid isomeric heterogeneity [18,82]. In general, matrix-assisted laser desorption ionization mass spectrometry imaging (MALDI-MSI) shows an excellent performance in the visualization of the spatial distribution of phospholipids and glycolipids, but the information of C=C location isomers cannot be acquired. To better obtain the information of C=C location isomers, some chemical derivatization methods were combined with MALDI-MSI. For instance, the PB reaction coupled with MALDI-MSI was used for the localization of the C=C bond in phospholipids and glycolipids within tissue samples, which provides a novel tool for investigating the biological roles of lipid isomers [83]. As a significant advance in capabilities for isomer-resolved MSI lipidomics, the recent study reported an implementation of isomer-resolved MALDI-MSI on a SYNAPT HDMS G2-Si quadrupole time-of-flight (Q-TOF) mass spectrometer, allowing for fast OzID for the imaging of both the C=C bond and sn-positional isomers in tissue samples [84]. Additionally, combining MALDI-MSI with OzID demonstrated that both the C=C bond and sn-positional isomeric lipids differed in spatial locations within mouse brain tissue sections [18]. On-tissue ozonization combined with MALDI-MSI enabled the imaging of the differential distribution of phospholipids with C=C bond positional isomers in tissue sections of mouse brain and human colon [85]. Similarly, by using *N*,*N*,*N*-trimethyl-2-(piperazin-1-yl)ethan-1-aminium iodide (TMPA), 20 FAs were successfully imaged in rat kidney and brain tissues by MALDI-MSI [86]. Furthermore, the performance of on-tissue PAA epoxidation combined with a MALDI-TOF/TOF-MS platform to map the spatial distribution of FA isomers within tumor tissue sections from a murine melanoma model has been confirmed (Figure 8A) [5]. The results provide evidence that the on-tissue epoxidation reaction coupled with the MALDI-TOF/TOF-MS platform could provide a high precision spatial distribution of FA isomers from complex tissue samples.

Additionally, DESI-MS combined with 193 nm UVPD could map the difference in lipid isomer compositions in the tissue sections (including mouse brain tissue section, human brain tissue section, and lymph node tissue section with thyroid cancer metastasis), as well as between tissue subtypes (e.g., the white and gray matter) (Figure 8B) [87] and could directly evaluate C=C positions in FFAs from biological tissue sections based on a charge-inversion strategy [21]. Furthermore, *m*-CPBA coupling with DESI-MS enabled a mapping of the alterations of lipid C=C isomers on tissue sections [25], suggesting that lipid C=C isomers were differentially expressed in distinct tissue regions and could be utilized to determine the tumor margin. In particular, when coupling the ^1^O_2_ reaction with nano-DESI-MS, spatial localization information of lipids positional isomers was readily obtained by MS^2^I [2]. Although the appealing advantages of AIMS promote the spatial visualization of unsaturated lipid isomers within various biological tissue samples, the low sensitivity of AIMS caused by a low ionization and transmission efficiency remains the main drawback that hinders the widespread application of AIMS in lipid imaging. In this regard, more and more effective chemical derivatization approaches will need to be developed [88].

### 3.5. Direct Characterization of Unsaturated Lipid Isomers in Other Samples

With the development of MS-based lipidomic tools, the biological samples used for the advanced lipidomic studies involved, but are not limited, to tissue, biofluid, and cell samples. For instance, the elucidation of the C=C bond position in FAs was rapidly profiled by paired offline ozonolysis with DART-MS in FAs extracts of *Hawaiian Drosophila* species. The results revealed that the unsaturation profile acquired from FAs extracts was helpful for the rapid differentiation of animals fed on different diets or animal species [64]. In addition, SCP-PB-nanoESI-MS was used to investigate the lipid compositions in lipid droplets of *Perilla seed* [47], whereas in situ ambient ionization by LTP can be used to determine C=C positions in unsaturated FAs and microbial fatty acid ethyl ester (FAEE) in *Salmonella enterica* Typhimurium INSP24 (SARA1) [54]. Additionally, on-demand electrochemical epoxidation incorporated with nanoESI-MS has been used for the analysis of complex lipids containing multiple C=C bonds in a natural lipid extract from chicken egg yolk [58]. The established method has features including a high throughput, low sample consumption, and an in-situ derivatization and characterization of the C=C bond, opening a new possibility for designing on-demand and specialized methods in nanoESI-MS in order to perform targeted analysis. Furthermore, the method where HAuCl_4_ was added into an electrospray solvent during microdroplet mass spectrometry analysis has been successfully applied to pinpoint the C=C bond in four unsaturated fatty acids (including linoleic acid (LA), ricinoleic acid (RA), isooleic acid (IA), and nervonic acid (NA)) and two phospholipids (including 1,2-dioleoyl-sn-glycero-3-phosphocholine (DOPC) and L-α-lysophosphatidylcholine (lysoPC)) [53]. Together, Table 1 highlights the applications of AIMS techniques in the elucidation of unsaturated lipids based on different derivatization strategies.

## 4. Conclusions and Future Directions

Profiling the complete molecular composition of lipidome is helpful for revealing the potential pathways and mechanism in lipid metabolism. Compared with traditional chromatographic separation coupled with MS, AIMS offers simplicity and a higher throughput for lipid structural elucidation. To date, a series of derivatization methods (including the PB reaction, OzID, epoxidation reaction, ^1^O_2_, UVPD, and plasma/electrochemical-induced epoxidation) in conjunction with various AIMS techniques (e.g., nanoESI-MS, DESI-MS/nano-DESI-MS, IR-MALDESI, and LTP-MS) have been successfully implemented for the direct structural characterization of lipid isomers from different biological samples (e.g., tissue, biofluids, and cell samples), showing great potential in lipid structural elucidation, both in laboratory research and clinical molecular diagnosis. Even though the field of lipidomics has been greatly advanced by AIMS, there remains challenges related to comprehensive lipidome identification and structural characterization.

First, owing to the highly complexity of the lipidome, besides the investigation of C=C positions, many more efforts are still needed for the elucidation of sn-positions, branched chain isomers, and the spatial distribution of broad types of unsaturated lipids. Additionally, a method that could be used for in situ and in vivo monitoring and offering an absolute quantitation of unsaturated lipid changes with high precision is still lacking. Current AIMS experiments have been carried out under ambient conditions, which are susceptible to interference from the environment. Hence, the reproducibility of the quantitative and qualitative analysis of unsaturated lipids in complex biological samples by AIMS need to be further improved. This might be improved either by the addition of internal standards or by using a commercial and high precision AIMS device, but many more efforts need to be made on method automation, hardware integration, and software development. Moreover, it is necessary to develop advanced computational tools, including automated software and machine learning algorithms, to facilitate the analysis of large datasets collected by different AIMS techniques coupled with derivatization methods. Ultimately, orthogonal and parallel biological studies need to be conducted to fully understand the functional implications caused by lipid structure heterogeneity and alteration.

## Figures and Tables

**Table 1:** The applications of AIMS techniques in elucidation of unsaturated lipids based on different derivatization strategies.

Derivatization Strategies	Reagents	AIMS Techniques	Sample	Analytes	LOD	Ref.
PB	Acetone or acetonitrile solution containing benzophenone	nanoESI-MS	Single cells	FAs, SMs, and PCs	0.1 pM	[6]
	Acetone	LMJ-SSP-MS	The sections of rat brain, lung, liver, spleen, and kidney, as well as in normal and diseased rat tissues	FAs and PCs	N/A	[67]
		nanoESI-MS	Rat brain tissue and rat organ tissues (including kidney, liver, and muscle), normal and cancerous mouse breast tissues	FAs, PEs, LPEs, LPSs, PIs, LPIs, PCs, LPCs, and GPs	N/A	[16]
			Rat brain, kidney, and liver tissues	FAs, PAs, LPAs, PGs, PSs, LPGs, PEs, and PIs	N/A	[68]
			Human plasma, whole blood, and cell lines	FAs	15 nM	[71]
			Zebrafish embryos	PCs	N/A	[69]
			Bovine blood, human blood, rat blood, and homogenized mouse brain samples	FAs, PEs, PSs, and PCs	N/A	[48]
	Benzophenone	SCP-nanoESI-MS	*Perilla seed*, Zebrafish muscle, and human intestinal tissue	FAs, PCs, and TAGs	N/A	[47]
	CH_3_OH/CH_2_Cl_2_/H_2_O (*v*/*v*/*v* = 8:1:1) containing 0.4 mg/mL benzophenone and 0.4 mg/mL NH_4_HCO_3_	ICE-nanoESI-MS	Human colorectal cancer tumors and paired distal noncancerous tissues	PCs, LPs, SMs, FAs, PAs, LPAs, PSs, LPSs, PGs, LPGs, PIs, LPIs, PEs, LPEs, CLs, and Cers	N/A	[70]
OzID	Ozone	DART-MS	*Hawaiian Drosophila species*	FAs	N/A	[64]
Epoxidation	peracetic acid	nanoESI-MS	(HPDE/E6E7) cells and (PANC-1) cells	FAs	4.2 nM	[5]
	*m*-CPBA	IR-MALDESI	Tissue sections of rat liver and human bladder	FAs	2–4 fmol per laser spot	[65]
		DESI-MS	Mouse kidney and metastatic lung tissue	FAs and PGs	10~100 pmole	[25]
	HAuCl_4_	microdroplet-MS	Standards	LA, RA, IA, NA, DOPC, and lysoPC	N/A	[53]
Electro-epoxidation	Hydrochloric acid and an acetonitrile/water	nanoESI-MS	Chicken egg yolk	PGs, PSs, and FAs	10 nM	[58]
Electro-oxidation	Ir and Ru		Serum	FAs	1.18–8.00 μM	[74]
Oxidation	^1^O_2_	nano-DESI-MS	Rat brain, mouse uterine, and gastrocnemius muscle tissue	FAs, LPEs, PEs, and PCs	N/A	[2]
UVPD	193 nm	DESI-MS	Mouse brain and kidney tissues, pancreas, kidney, lung, fallopian, ovarian tissue, and endometrial tissue	PCs	∼500 nM	[87]
			Mouse brain tissue, human ovarian tumor tissue, and breast cancer tissue	FAs	N/A	[21]
LTP	Pure helium	nanoESI-MS	Human plasma	FAs	0.07 μM	[72]
	Oxygen		Bovine liver polar extract	PCs, PAs, PEs, PGs, and PIs	N/A	[73]
	Reactive oxygen species in the plasma	LTP-MS	*Human, Equine, and Fetal Bovine Serums*	FAs	0.1 μM	[55]
	Ozone		*Salmonella enterica* typhimurium INSP24 (SARA1)	FAs and FAEE	N/A	[54]

N/A represents information not available.

## Abbreviations

Ambient ionization mass spectrometry (AIMS), Paternò–Büchi (PB) reaction, ozone-induced dissociation (OzID), carbon–carbon double bond (C=C), type II diabetes (T2D), mass spectrometry (MS), tandem mass spectrometry (MS/MS), collision-induced desorption (CID), electron-induced dissociation (EID), ultraviolet photodissociation (UVPD), meta-chloroperoxybenzoic aid (*m*-CPBA) epoxidation, peracetic acid (PAA) epoxidation, liquid chromatography (LC), desorption electrospray ionization mass spectrometry (DESI-MS), rapid evaporative ionization mass spectrometry (REIMS), internal extractive electrospray ionization mass spectrometry (iEESI-MS), atmospheric pressure matrix-assisted laser/desorption ionization mass spectrometry (AP-MALDI-MS), matrix-assisted laser desorption/ionization mass spectrometry (MALDI-MS), PB reaction coupling to tandem MS (PB-MS/MS), 2-acetylpyridine (2-AP), 2′,4′,6′-trifluoroacetophenone (triFAP), *N*,*N*-diethyl-1,2-ethanediamine (DEEA), free fatty acids (FFAs), polymer coating transfer enrichment (PCTE), PB reaction with surface-coated probe nanoESI-MS (SCP-PB-nanoESI-MS), matrix-assisted laser desorption/ionization time-of-fight/time-of-fight MS (MALDI-TOF/TOF-MS), fatty acids (FAs), limit of detections (LODs), relative standard deviations (RSDs), chloroauric acid (HAuCl_4_), low-temperature plasma (LTP), paper-based analytical devices (PADs), singlet oxygen (^1^O_2_), lipid hydroperoxides (LOOHs), mass spectrometry imaging (MSI), nanospray desorption electrospray ionization mass spectrometry (nano-DESI-MS), ion mobility-mass spectrometry (IM-MS), high-performance liquid chromatography-tandem mass spectrometry (HPLC-MS/MS), glycerophospholipids (GPs), liquid microjunction surface sampling probe (LMJ-SSP), phosphatidylserines (PSs), phosphatidylglycerols (PGs), phosphatidylinositols (PIs), phosphatidylethanolamines (PEs), phosphatidic acids (PAs), lyso-PAs (LPAs) and lyso-PGs (LPGs), nonalcoholic fatty liver disease (NAFLD), infrared matrix-assisted laser desorption electrospray ionization (IR-MALDESI), in-capillary extraction nanoelectrospray ionization mass spectrometry (ICE-nanoESI-MS), phosphatidylcholines (PCs), phosphatidylinositols (PIs), neutral loss scan (NLS), point-of-care (POC), HPV16-E6E7 immortalized human pancreatic duct epithelial (HPDE/E6E7) cells, pancreatic cancer cells (PANC-1), single cell mass spectrometry (SCMS), mass spectrometry imaging (MSI), matrix-assisted laser desorption ionization mass spectrometry imaging (MAL-DI-MSI), quadrupole time-of-flight (Q-TOF), *N*,*N*,*N*-trimethyl-2-(piperazin-1-yl)ethan-1-aminium iodide (TMPA), fatty acid ethyl ester (FAEE), *Salmonella enterica* Typhimurium INSP24 (SARA1), lysophosphatidylcholines (LPCs), sphingomyelins (SMs), linoleic acid (LA), ricinoleic acid (RA), isooleic acid (IA), nervonic acid (NA), 1,2-dioleoyl-sn-glycero-3-phosphocholine (DOPC), L-α-lysophosphatidylcholine (lysoPC), lysophosphatidylserines (LPSs), lysophosphatidylinositols (LPIs), lysophosphoethanolamines (LPEs), cardiolipin (CLs), and ceramides (Cers).
